# Frequency and Causes of Hypotonia in Neonatal Period with the Gestational Age of More Than 36 Weeks in NICU of Mofid Children Hospital, Tehran, Iran During 2012-2014

**Published:** 2017

**Authors:** Nosratollah Seyed SHAHABI, Hossain FAKHRAEE, Mohammad KAZEMIAN, Abolfazl AFJEH, Minoo FALLAHI, Maryam SHARIATI, Fatemeh GORJI

**Affiliations:** 1 Neonatal Health Research Center (NHRC), Shahid Beheshti University of Medical Sciences, Tehran, Iran

**Keywords:** Neonatal hypotonia, Genetic, Peripheral, Central

## Abstract

**Objective:**

Hypotonia is a serious neurologic problem in neonatal period. Although hypotonia is a nonspecific clinical finding but it is the most common motor disorder in the newborn. The objective of this study was to determine the frequency of neonatal hypotonia then to ascertain of the most common causes.

**Materials & Methods:**

This cross –sectional prospective study was carried out on the 3281 term infants hospitalized in conventional and NICU of Mofid Children Hospital, Tehran, Iran during 2012-2014. Diagnosis was made by history, physical & neurological examination and accessible diagnostic tests.

**Results:**

Fifty nine hypotonic neonates were identified, forty seven (79.66%) had central hypotonia (Hypoxic ischemic encephalopathy (n= 2), other causes of encephalopathy (n=2), intracranial hemorrhage (n=4), CNS abnormalities (n= 7), chromosomal disorders (n=4), syndromic–nonsyndromic (n=8), and metabolic diseases (n=8). Peripheral hypotonic recognized in 6 infants (10.17%); spinal muscular atrophy (n= 1), and myopathy (n= 5). Six cases (10.17%) remained unclassified. Twelve infants had transient hypotonia. In final study, 18 of 59 infants (30%) died, nearly 90% before one year of age. Twenty-eight (47%) infants found developmental disorders and only 13 (22%) infants achieved normal development in their follow up.

**Conclusion:**

Neonatal hypotonia is a common event in neonatal period. A majority of diagnosis is obtained by history and physical examination. Neuroimaging, genetic and metabolic tests were also important in diagnosis. Genetic, syndromic–nonsyndromic, and metabolic disorders were the most causes of neonatal hypotonia.

## Introduction

Muscle tone is the amount of tension or resistance to movement in a muscle. Normal tone requires an integration of central and peripheral nervous system function. Although hypotonia is a nonspecific clinical sign but accounts the most common motor disorder in the neonatal period. First, we should try to classify hypotonia into the central and peripheral type though; this is often hard to do with definitely. 

The presence of decreased state of consciousness, seizure, dysmorphic feature and malformation in other organs, normal or brisk deep tendon reflexes suggest to central origin, on the other hand the presence of alertness, severe weakness, absence of deep tendon reflexes and reduced antigravity movements are criteria that mostly seen in involvement of the peripheral nervous system (1, 2). 

Arthrogryposis especially in distal limbs, dislocation of hip joints, hypofunction of intrauterine respiration which manifestation of it produce deformity of thorax and hypoplastic lung revealed that hypotonia had already been in intrauterine. Most type of neonatal hypotonia is central origin and only less of them belong to the peripheral type (2-6). 

A significant cause of hypotonia in infants and children is the presence of one of the genetic syndromes associated with hypotonia. In nonsyndromic hypotonia; patients do not have a recognizable collection of somatic dysmorphic features but still have anomalies or abnormalities in the CNS that result in hypotonia (7). 

Congenital myopathies are not rare in the neonatal period and account for 20% of hypotonia at birth and clinical work up may require muscle enzyme studies and /or muscle biopsy (8). Many genetic and metabolic conditions may present with signs of hypotonia, the mechanisms by which these disorders affect muscle tone are varied and depend upon the underlying gene defect (9). 

The objective of present study was to determine the frequency and most common causes of neonatal hypotonia.

## Materials & Methods

This cross sectional prospective study was carried out in 2012-2014 at Neonatal Department & NICU in Mofid Children Hospital, Tehran, Iran. Parental consanguinity, maternal illness, drug consumption during pregnancy, type of delivery, Apgar score, vital sign, weight of birth, causes of admission, feeding difficulties, respiratory problems, need for ventilator, inserted in especial form that had been prepared by one of author. 

Informed consent was taken from the subjects parents and the study was approved by local Ethics Committee. Inclusion criteria included age of the patient less than one month during admission and hypotonia more than two weeks. Exclusion criteria were hypoglycemia, trauma or toxic exposure, and those who had sepsis or cardiopulmonary diseases. 

Details of history and physical examination, including muscle tone, DTR, dysmorphic features of face or other abnormalities were noted. Diagnostic tests were selected on the location of CNS involvement; neonates with decrease state of consciousness, dysmorphic features, seizures, and normal or brisk DTRs were placed on central type hypotonia, such as hypoxic ischemic brain encephalopathy or other causes of encephalopathy, intracranial hemorrhage, chromosomal disorders, syndromic-nonsyndromic disorders, metabolic disorders and CNS malformations need neuroimaging, EEG, specific tests for metabolic disorders, karyotype analysis, genetic tests. 

Alert infants who had severe muscular weakness, reduced antigravity movements were considered in the peripheral group; motor neuron disease, peripheral neuropathy, and myopathies, demanding, EMG, muscle enzyme, muscle biopsy. 

Data were analyzed using SPSS 18 (Chicago, IL, USA).

## Results

Of 3281 term and near term infants admitted from 2012-2014 in conventional and NICU, 59 (1.18%) had hypotonia. Fifty-nine infants met the inclusion criteria, 39 (66.1%) infants were male and 20 female (Ratio = 2:1). Parental consanguinity was presented in 21 cases (35.6%). Forty infants (67.8%) were delivered by cesarean section and 19 by vaginal route. Fourteen mothers (23.7%) had hypothyroid and all were on levothyroxine during pregnancy. Polyhydramnios were reported in seven pregnancies (Table 1). 

**Table 1:** Common Symptoms in Mothers During Pregnancy

**Common symptoms**	**Central** **No.: 47**	**Peripheral** **No.:6**	**unknown** **No.:6**	**Total** **No.:59**
**Hypothyroidism**	10(71.4%)	3(21.4%)	1(7.2%)	14(23.7%)
**Polyhydramnios**	4(57.1%)	2(28.6%)	1(14.3%)	7 (11.9%)
**Breech presentation**	4(80%)	1(20%)	0(0%)	5(8.5%)
**Bleeding during pregnancy**	3(100%)	0(0%)	0(0%)	3(5.1%)

Low Apgar score were noted in 9 infants (six in central group, one in peripheral and two in unknown type). Most of the patients referred for admission had difficulties in feeding or respiratory distress symptoms but in careful neurologic examination, their main problems were underlying neurologic conditions. Feeding difficulties were present in 31 infants (52.5%); uttermost had swallowing difficulties that necessitated using of nasogastric tube and in a small number of them led to gastrostomy. Respiratory distresses were present in 17 cases, more than 70% of peripheral type and 30% of central type hypotonic infants required intubation and ventilation in the first days or weeks of admission. Nine patients had both feeding and respiratory problems. 

**Table 2:** Characteristics of Hypotonic Infants

**Common symptoms**	**Central** **No. (%)**	**Peripheral** **No. (%)**	**Unknown No. (%)**	**Total** **No. (%)**
**Consanguinity**	17 (81)	2 (9.5)	2 (9.5)	21 (35.6)
**C- section**	31 (77.5)	4 (10)	5 (12.5)	40 (67.8)
**Male**	32(82.1)	5(12.8)	2 ( 5.1)	39 (66.1)
***Apgar *** **>6** ***Apgar *** **<6** ***Apgar: *** **Unavailable**	30 (83.33) 6 (66.7) 11 (78.6)	3 (8.33) 1 (11.1) 2 (14.3)	3 (8.33) 2 (22.2) 1 (7.1)	36 (61.0) 9 (15.3) 14 (23.7)
**Nutritional problems**	22 (71)	5 (16.1)	4 (12.9)	31 (52.5)
**Respiratory problems**	10 (58.8)	4 (23.5)	3 (17.7)	17 (28.8)
**Dysmorphic features**	8 (100)	0 (0)	0 (0)	8 (13.6)
**Seizure**	6 (75)	0 (0)	2 (25)	8 (13.6)
**Lethargy**	5 (83.3)	0 (0)	1 ( 16.7)	6 (10.2)
**Laryngomalacia**	2 (50)	1 (25)	1 (25)	4 (6.8)
**Normal development**	12 (92.3)	0 (0)	1 (7.69)	13 (22)
**Developmental delay**	20 (74.1)	4 (14.8)	3 (11.1)	28 (47)
**Death**	15 (83.3)	2 ( 11.1)	1 (5.5)	18 (30.5)

Dysmorphic features more often increase the likelihood of CNS dysfunction as an example for hypotonia, dysmorphic features recognized in 8 infants, all belonged to central group. Seizures occurred in eight patients and were more frequent in central group hypotonia (Table 2). Among 47 cases diagnosed as central hypotonia there were hypoxic ischemic insult in (n=2), other causes of encephalopathy in (n=2), intracranial hemorrhage in (n=4), CNS malformation in (n=7), and Down syndrome in (n=4). Prader-Willi Syndrome was diagnosed in 3 infants (one confirmed and two suspected), nonsyndromic in (n=5), and metabolic disorders in (n=8) patients. In fact, one patient had neurometabolic disorder distinguished as “ Zellweger disease”. 

Twelve infants had transient hypotonia; were included in central group because they had history of seizures and were lethargic when admission just diagnostic tests revealed nothing abnormal. They achieved with their developmental milestone in a few months later. 

Six infants had peripheral hypotonia and one of them was distinguished Werdnig Hoffmann disease but the remaining clinically were diagnosed as myopathy. In fact, patients had almost clinical sign of typical myopathic conditions including myopathic faces with paucity of facial expression, as well as high arch palate on the other hand they did not have decrease state of responsiveness or poor visual contact. All investigational examinations were in the range of normal. No diagnosis was identified in six hypotonic infants (Table 3). 

**Table 3:** Summary of Diagnosis in Hypotonic Infants

**Central hypotonia**	**47 (79.66%)**
Hypoxic-ischemic	**2 (4.26)**
Other causes of encephalopathy	**2 (4.26)**
cerebral haemorrhage	**4 (8.51)**
Cerebral anomalies	**7 (14.89)**
Chromosomal abnormalities	**4 (8.51)**
Syndromic hypotonia*	**3 (6.38)**
Non syndromic hypotonia	**5 (10.64)**
Metabolic disorders**	**8 (17.2)**
Transient hypotonia	**12 (25.53)**
**Peripheral hypotonia**	**6 (10.17)**
Spinal Muscular Atrophy	**1 (16.7)**
Myopathy	**5 (83.3)**
**Undiagnosed Hypotonia**	**6 (10.17)**

* Prader Willi Syndrome confirmed in one & suspected in two

** Neurometabolic (Zellweger disease) in one case

The outcome of infants who were admitted due to hypotnoia and followed for two years deserves consideration. In the peripheral group, two patients died and four had solitary motor delay. Thirteen (22%) infants achieved normal development, 12 were in central group and one in unclassified. Neurodevelopmental delay observed in 28 infants (47%), most of them had global developmental delay. Eighteen (30%) infants died at the end of study, 88%occurred before their first year of age and 50% happened in younger than their six months old. Two of 18 infants had a peripheral hypotonia. 

Neuroimaging made a specific contribution to diagnosis. CT scan/ MRI were performed in 28 infants; abnormalities were seen in 17 cases. Three patients had clinical signs of Prader Willi syndrome, of these one confirmed by fluorescence in situ hybridization test.

EMG /NCV test revealed Werdnig Hoffmann disease in one case and myopathic change in another. EMG/ NCV results were normal in the rest of peripheral type hypotonia. Metabolic tests were carried out in those infants clinically present with lethargy, hypotonia, unexplained acidosis, hepatomegaly, seizures, and hypoglycemia. Specific tests was considered including serum & urine amino acids, serum ammonia, lactate, and pyruvate and urine organic acids, urinary reducing substance. The results of diagnostic tests are summarized in Table 4. 

**Table 4:** Results of Diagnostic Tests

**Diagnostic test**	**Normal**			**Abnormal**			**Total**	
	**Central**	**Peripheral**	**Unknown**	**Central**	**Peripheral**	**Unknown**	**Normal**	**Abnormal**
**Neuroimaging**	6	2	3	17	0	0	11	17
**EEG**	4	1	2	1	0	0	7	1
**EMG**	4	1	1	0	1	0	6	1
**Muscle biopsy**	0	1	1	0	0	0	2	0
**Karyotype**	1	0	0	0	0	0	1	0
**Metabolic test** *	1	2	1	7	0	0	4	7
**FISH TEST** **	0	0	0	1	0	0	1	0

* Metabolic test results: Lucine =2 cases, Ammonia =2 cases, Citruline =1 case, Lactate & Isovaleric acid =1 case, Lactate&Ammonia =1 case, CSF Glycine =1case

** Fluorescence in Situ Hybridizidation

## Discussion

Hypotonia is a common sign of motor system dysfunction in the newborn may be caused by a lesion at any level of neuronal axis. There are various approaches to evaluation of floppy baby. Localization of the lesion is a useful exercise in neurological examination. Either those conditions that affect the brain and brainstem, diffusely or focally is called supraspinal or suprasegmental conditions. 

Those conditions that involve the motor unit are named segmental or motor unit conditions (10). A clinical examination could help to differentiate hypotonia with weakness (paralytic) from hypotonia without weakness (non paralytic) (11). In paralytic type or peripheral hypotonia, there is profound weakness and deep tendon reflexes are usually lost (11). Generally, the proportion of central hypotonia is much more than the peripheral type. The present prospective study demonstrated that this proportion was more than 5:1 ratio. In a study, 66% of hypotonia were central type and 34% had peripheral origin (6). Durja Paro Panjanand et al. studied on 138 neonate with hypotonia during 10 years, most common causes of hypotonia were chromosomal, syndromic and metabolic disorders, and found 121 (88%) patients with central hypotonia and 13 (9%) with peripheral and 4 (3%) infants were unclassified (7). 

In our study 79.6% (n=47) of hypotonia related to central type, 10.6% (n=6) to peripheral type and 10.6% (n=6) to infants that remained undiagnosed. Jimenez et al. conducted a systematic review of newborn infants with hypotonia. A total of 73 hypotonic newborn infants were identified, 21 (28.7%) of whom met eligibility criteria. The majority 17 (81%) were classified on central hypotonia that was chromosomal disorders 8 (47%), metabolic disorders 5 (29.4%), cerebral malformations 4 (23.5%) and the remaining 4 (19%) were graded as peripheral hypotonia (12). Results of our study is similar to the Jimenez et al. study, since we found out chromosomal disorders (n=4) 6.8%, syndromic –nonsyndromic (n=8) 13.6%, cerebral malformations (n=7) 11.9%, and metabolic disorders (n= 8) 13.6% (12). 

In other studies, hypoxic ischemic encephalopathy was the most common causes of neonatal hypotonia. There is no specific test for hypoxic ischemic encephalopathy, the disorder diagnosed on the basis of clinical manifestation, birth history, and neuroimaging evidence (2, 6). Our study and in two other assessments (7, 12) demonstrate that genetic - syndromic and metabolic disorders are the most common causes. 

Maternal hypothyroidism found in 14 (23.7%) cases. Whether maternal hypothyroidism could play a role in the causes of neonatal hypotonia or not is doubtful ? History and physical examination is the main program in diagnosis of neonatal hypotonia. Despite substantial advance in neuroimaging and laboratory studies technology cannot replace the clinicians’ anatomical localization based on history and physical examination. Paro–Panjan with consideration of history and detail clinical finding and task forth dysmorphology could distinguish hypotonia in neonatal period in fifty nine percent. 25.5% of neonatal hypotonia diagnosed by neuroimaging, biochemical and genetic tests, 6% of them recognized with neurophysiology, special molecular test and muscle biopsy (7). 

We recognized 55.9% of neonatal hypotonia by taking history and physical examination. 28.8% of diagnoses were obtained by neuroimaging and 11.9% from genetic and metabolic tests. There is similarity between the two studies, except for undetermined neonatal hypotonia that was 2.9% in Paro –Panjan’s research and 10.2% in our study. The number of our cases in two years is much more than presented by Jimenze’s research during a prolonged time (12).The mortality rate in our study was 30% and the same result as reported earlier (2). We found out a large number of hypotonic infants in short time. It will be instructive if this study is performed in long term.

**In conclusion,** hypotonia presenting in the first days or weeks of life should alert the neonatologist because of the serious potentially underlying condition. The more common causes of hypotonia in our study were cerebral malformations and genetic–metabolic disorders. More than 50% of diagnosis could be identified by careful history and physical examination. Neuroimaging is the next most importance in diagnosis. If the onset of hypotonia is in the first days of birth, with prolonged feeding and respiratory difficulties, the prognosis is poor and associated with high rate of mortality and morbidity. Genetic counseling is essential.

## References

[1] Miller VS, Delgado M, Iannaccone ST ( 1993). Neonatal hypotonia Seminar in neurology.

[2] Laugal V, Cossee M MJ, de Saint –Martin A, Echaniz- Laguna A, Mandel JL, Astruc D, Messer FMJ (2008). Diagnostic approach to neonatal hypotonia: retrospective study on144neonates. Eur J Pediatr.

[3] Birdi K, Prasad C, Chodirker B, Chudly AE (2005). The floppy infant: retrospective analysis of clinical experience (1990-2000) in a tertiary care facility. J Chlid Neurol.

[4] Johnston HM (2003). The floppy weak infant revisited. Brain Dev.

[5] Crawford TO (1992). Clinical Evaluation of the Floppy infant. Pediatric Annal.

[6] Richer LP, Shevell MI, Miller SP (2001). Diagnostic profile of neonatal hypotonia; An 11 year study. Peditric Neurol.

[7] Paro–Panjan D (2004). Congenital hypotonia is there an algorithm. J Child Neurology.

[8] Griggs RC, Mendell JR, Miller RG (1995). Cngenital myopathies. Evaluation and treatment of myopathies.

[9] Nada Zadeh, Louanne Hudgings (2009). The Genetic Approach to hypotonia in the neonate. NeoReviews.

[10] Bodenstiener JB (2008). The evaluation of the hypotonic infant Seminar in PediatricNeurology.

[11] Dubowitz V, Thomas NH (1994). The natural history of type 1(severe) spinal muscular dystrophy. Neuromuscular Disord.

[12] Jimenez E, Garcia – Cazoria A, Colomer J, Nascimento A, Ieiondo M, Compistol J (2013 ). Hypotonia in the neonatal period: 12 years experience [Article in Spanish]. Rev Neurol.

